# Contactless Monitoring System Versus Gold Standard for Respiratory Rate Monitoring in Emergency Department Patients: Pilot Comparison Study

**DOI:** 10.2196/44717

**Published:** 2024-02-16

**Authors:** Charlotte E Goldfine, Md Farhan Tasnim Oshim, Brittany P Chapman, Deepak Ganesan, Tauhidur Rahman, Stephanie P Carreiro

**Affiliations:** 1 Division of Medical Toxicology Department of Emergency Medicine Brigham and Women's Hospital Boston, MA United States; 2 Manning College of Information and Computer Sciences University of Massachusetts Amherst Amherst, MA United States; 3 Department of Emergency Medicine University of Massachusetts Chan Medical School Worcester, MA United States; 4 Halıcıoğlu Data Science Institute University of California San Diego San Diego, CA United States

**Keywords:** cardiopulmonary monitoring, contactless monitor, radar, respiratory rate, vital signs

## Abstract

**Background:**

Respiratory rate is a crucial indicator of disease severity yet is the most neglected vital sign. Subtle changes in respiratory rate may be the first sign of clinical deterioration in a variety of disease states. Current methods of respiratory rate monitoring are labor-intensive and sensitive to motion artifacts, which often leads to inaccurate readings or underreporting; therefore, new methods of respiratory monitoring are needed. The PulsON 440 (P440; TSDR Ultra Wideband Radios and Radars) radar module is a contactless sensor that uses an ultrawideband impulse radar to detect respiratory rate. It has previously demonstrated accuracy in a laboratory setting and may be a useful alternative for contactless respiratory monitoring in clinical settings; however, it has not yet been validated in a clinical setting.

**Objective:**

The goal of this study was to (1) compare the P440 radar module to gold standard manual respiratory rate monitoring and standard of care telemetry respiratory monitoring through transthoracic impedance plethysmography and (2) compare the P440 radar to gold standard measurements of respiratory rate in subgroups based on sex and disease state.

**Methods:**

This was a pilot study of adults aged 18 years or older being monitored in the emergency department. Participants were monitored with the P440 radar module for 2 hours and had gold standard (manual respiratory counting) and standard of care (telemetry) respiratory rates recorded at 15-minute intervals during that time. Respiratory rates between the P440, gold standard, and standard telemetry were compared using Bland-Altman plots and intraclass correlation coefficients.

**Results:**

A total of 14 participants were enrolled in the study. The P440 and gold standard Bland-Altman analysis showed a bias of –0.76 (–11.16 to 9.65) and an intraclass correlation coefficient of 0.38 (95% CI 0.06-0.60). The P440 and gold standard had the best agreement at normal physiologic respiratory rates. There was no change in agreement between the P440 and the gold standard when grouped by admitting diagnosis or sex.

**Conclusions:**

Although the P440 did not have statistically significant agreement with gold standard respiratory rate monitoring, it did show a trend of increased agreement in the normal physiologic range, overestimating at low respiratory rates, and underestimating at high respiratory rates. This trend is important for adjusting future models to be able to accurately detect respiratory rates. Once validated, the contactless respiratory monitor provides a unique solution for monitoring patients in a variety of settings.

## Introduction

Respiratory rate is a fundamental vital sign that serves as an indicator of physiologic function [1]. Changes in respiratory rate are often one of the first indicators of severe illness and clinical deterioration in a variety of disease states, such as sepsis, metabolic acidosis, respiratory distress, and drug toxicities [2,3]. Early identification of clinical decline by recognition of changes in respiratory rate is associated with improved outcomes, such as decreased intensive care unit admissions, decreased length of stay, and improved functional outcomes in patients [4]. However, respiratory rate is often considered a “neglected vital sign” due to inconsistent documentation [5]. There are 2 techniques currently used to measure respiratory rate. The gold standard for determining respiratory rate is manual counting. The current standard of care in hospital facilities and emergency departments (EDs) is transthoracic impedance plethysmography, which measures chest wall movement through cardiac telemetry electrodes. However, both techniques have several limitations. Manual counting is labor-intensive, needs to be done by visually examining the patient, and cannot practically be continuous for long periods of time [6,7]. Transthoracic impedance plethysmography is susceptible to inaccurate readings, as any aberrant movement of the patient makes the monitoring ineffective [8,9].

The development of technologies that accurately and effectively monitor respiratory rates is important to improve the care of patients. Additionally, with the widespread use of telemedicine, digital health interventions that can be used in outpatient settings or settings where a patient cannot be closely monitored are increasingly needed. One such technology is a contactless sensor system that uses an ultrawideband impulse radar–based contactless respiratory monitor PulseON 440 (P440) capable of detecting subtle movements in participants (eg, as the chest wall rises). The P440 works by sending an electromagnetic wave with a transmitter antenna, the reflections of which are caught by the receiver antenna. The device uses 2-way time of flight ranging to measure the distance between the radar and a target. In addition, this radar is a coherent radio transceiver that allows the energy in each transmitted pulse to be summed, improving the signal-to-noise ratio of received transmissions. This monitor has demonstrated high accuracy in laboratory testing, is less than 2 cm in size, and can perform scanning at rates up to 125 Hz at distances up to 30 meters [10]. The monitor operates at approximately 50 μW, which is considered a very low power transmission. For comparison, a standard incandescent lightbulb operates at 60 W. Its capability to detect small movements with extreme accuracy makes this device optimally suited to measuring respiratory rate.

As new technologies are being developed, it is important to compare them to current gold standards to assess whether these technologies are both accurate and practical for clinical use. This study aimed to pilot-test the P440 radar in a cohort of ED patients to (1) compare the performance of the P440 radar to the gold standard (manual counting) and to the standard of care (transthoracic impedance plethysmography) in a clinical setting and (2) compare performance in subgroups of interest (patients with cardiopulmonary diagnoses and by sex).

## Methods

### Recruitment

This pilot study was performed at a large, academic, tertiary-care, level-one trauma center in central Massachusetts that treats approximately 135,000 patients per year. A convenience sample was enrolled during the study period (June 2019-February 2020). Eligible participants were aged 18 years or older, were being monitored by cardiac telemetry for respiratory rate in the ED, and were able to provide informed consent. Individuals were excluded if they were pregnant or were prisoners, as these populations are routinely excluded in this phase of research. Participants were screened for eligibility through the ED electronic medical record and approached for consent after discussion with the treating ED clinical providers.

### Study Design

**Figure 1:**
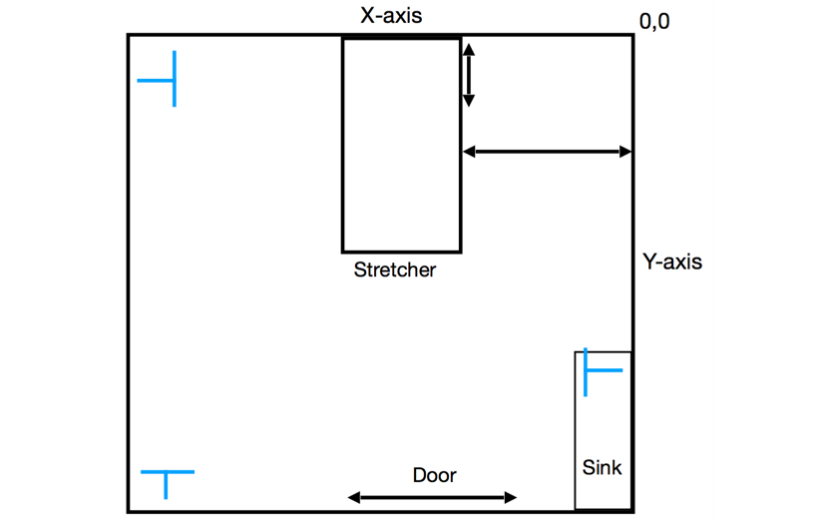
Schematic of patient room set-up. Blue Ts represent the PulsON 440 radar units.

Once the participant consented to participate, basic demographic and relevant clinical information were obtained from the electronic health record. A total of 3 P440 radar units were placed in a triangulated formation in the participant room for a 2-hour period (Figure 1). During the 2-hour period, the gold standard respiratory rate (manual counting) and the standard of care respiratory rate (transthoracic impedance plethysmography) were recorded at 15-minute intervals. Relevant clinical data (including medications given during the study period and significant events) were recorded.

### Hardware

**Figure 2:**
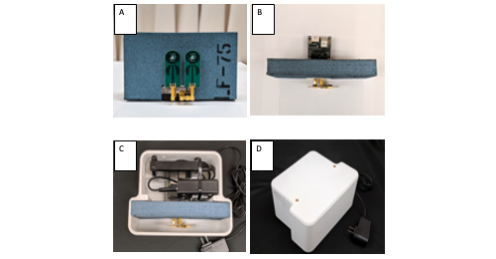
(A) PulsON 440 radar monitor with LF75 absorber behind the antenna. (B) Top view of the radar with absorber. (C) Radar box with Raspberry Pi and a hard disk drive. (D) Enclosed radar box.

The main study device was the P440, which used radar technology to calculate respiratory rate. Data were collected using application programming interfaces. The Raspberry Pi3 (Raspberry Pi Foundation) interfaced with the P440 (programed in C language) and stored all collected data on a microSD card. Each radar unit consisted of the P440 module, an absorber, a Raspberry Pi unit, and a hard disk (Figure 2). All components were assembled in a 3D-printed box. Details of the radar have already been published elsewhere [10].

### PulsON 440 Data Cleaning, Processing, and Fusion

The radar data were collected from all 3 devices in raw radargram format. The files were marked with Uniplexed Information Computing System (UNIX) timestamps and were synchronized in order to have the same event observed by all 3 radars for the same time windows. Raw radargrams contained information about participant movement as well as reflections from all the static clutter present in the environment (eg, walls, stretchers, and medical furniture). The first step in cleaning the raw data involved removing the static clutter [11]. A background subtraction technique was used to get rid of the clutter. The mean of the first 100 scans where there were no participants was subtracted from each window of 30 seconds. A bandpass filter was applied to remove direct current and high-frequency noise from the radargrams.

The system leveraged the different vantage points of the radars in 3 different positions in the room to observe the respiratory motion of the participant. As the orientation of the participant was not known and in a dynamic environment, we applied an independent component analysis (ICA) to find out which radar had the best respiratory signal [12].

Each radar signal and radargram were then filtered by an equiripple finite impulse response filter at cutoffs analogous to the normal breathing range from 6 to 30 breaths per minute. The location of the participant being monitored was extracted using a localization algorithm along with trilateration and Kalman filtering [13,14]. From each radar, the system focused on 50 range bins (45 cm) centered at the participants’ location bin. The focused range bin forms 50 individual time series of 30 seconds for a particular window. The 2D signal with 50 timeseries was then collapsed to a 1D signal using a sum-aligned function. The function found the timeseries that had the highest root-mean-square energy in that window and used it as a reference to align the remaining 49 timeseries with itself. After alignment, the function was summed to generate the global sum-aligned 1D signal, which preserved the breathing motion.

The ICA was applied to all 3 radars in 1D time series. The ICA output 3 independent components, which underwent a fast Fourier transform (FFT). The component with the strongest FFT peak was selected as the most dominant radar signal. We applied 2 different frequency detection methods to further identify the respiration rate from the selected radar—FFT and zero-crossing rate [10].

### Statistical Analysis

Descriptive statistics were calculated for basic demographic and clinical information. We compared the respiratory rate measured by the P440 with the gold standard (manual) respiratory rate by the Bland-Altman plot method using GraphPad Prism (version 9; GraphPad Software). This approach used the means and differences between the pairs of readings to calculate the mean difference (bias) and the upper and lower limits of agreement [15,16]. We considered an acceptable upper and lower limit of agreement to be –2 to 2 breaths per minute. We subsequently calculated the intraclass correlation coefficient (ICC) for the 2 methods of measurement. We then repeated this procedure for the gold standard (manual) and standard of care (transthoracic impedance plethysmography) respiratory rates. The Bland-Altman plot method was also used to compare respiratory rate differences found between the P440 and gold standard in participants based on sex and the presence of a cardiopulmonary diagnosis.

### Ethical Considerations

The study was approved by the University of Massachusetts Chan Medical School Institutional Review Board (protocol number H00016885). All participants provided informed consent. Study data were deidentified. Participants were not remunerated.

## Results

### Participant Data

A total of 14 participants were enrolled in the study. The demographics of the study participants are detailed in Table 1. The study population was comprised of 57% (8/14) male candidates with an average age of 58 years.

**Table 1:** Participant characteristics.

	Characteristics					Value
	Age (years), mean (range)					58.64 (23-86)
**Sex, n (%)**						
			Male	8 (57)		
			Female	6 (43)		
**Race, n (%)**						
		Asian			1 (7)	
		White			8 (57)	
		Declined to answer			4 (27)	
		Other			1 (7)	
**Hispanic or Latino, n (%)**						
		Yes			1 (7)	
		Unknown			1 (7)	
**Home medications, n (%)**						
			Opioid	2 (14)		
			Benzodiazepine	3 (21)		
**Admitting diagnosis, n (%)**						
			Gastrointestinal bleed	2 (14)		
			Diverticulitis	1 (7)		
			Sepsis	1 (7)		
			Hypoglycemia	1 (7)		
			Pancreatitis	1 (7)		
			Cardiac diagnoses^a^	6 (43)		
			Pulmonary diagnoses^b^	2 (14)		

^a^Cardiac diagnoses included chest pain, non-ST elevation myocardial infarction, and atrial fibrillation with rapid ventricular response.

^b^Pulmonary diagnoses include pneumonia and pulmonary edema.

### Comparison of Respiratory Rate Measurements

**Figure 3:**
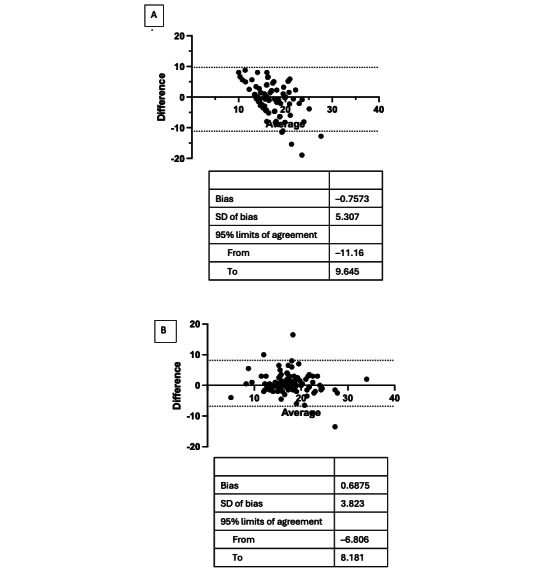
Bland-Altman plot for (A) PulsON 440 (P440) and gold standard and (B) gold standard and standard of care.

The P440 and gold standard Bland-Altman analyses showed a bias of –0.76. The upper limit of agreement was 9.65, and the lower limit of agreement was –11.16 (Figure 3A). At lower respiratory rates, the bias was more positive, indicating a higher measured respiratory rate by the P440 than the gold standard. At higher respiratory rates, the bias was more negative, indicating a lower measured respiratory rate by the P440 than the gold standard. About 34% (30/88) of the differences in measurements were within the prespecified clinically significant limits of agreement of –2 to 2. The P440 and gold standard respiratory rates did not have a statistically significant correlation. The ICC was 0.38 (95% CI 0.06-0.60). The mean absolute error between the P440 and gold stand respiratory rates was an average of 3.89 (range 1.55-8.51).

The standard of care and the gold standard Bland-Altman analysis showed a bias of 0.69. The upper limit of agreement was 8.18, and the lower limit of agreement was –6.81 (Figure 3B). Around 59% (52/88) of the differences in measurements were within the prespecified clinically significant limits of agreement of –2 to 2. The gold standard and standard of care respiratory measurements did have a statistically significant correlation. The ICC was 0.82 (95% CI 0.72-0.88).

### Subgroup Analyses

**Figure 4:**
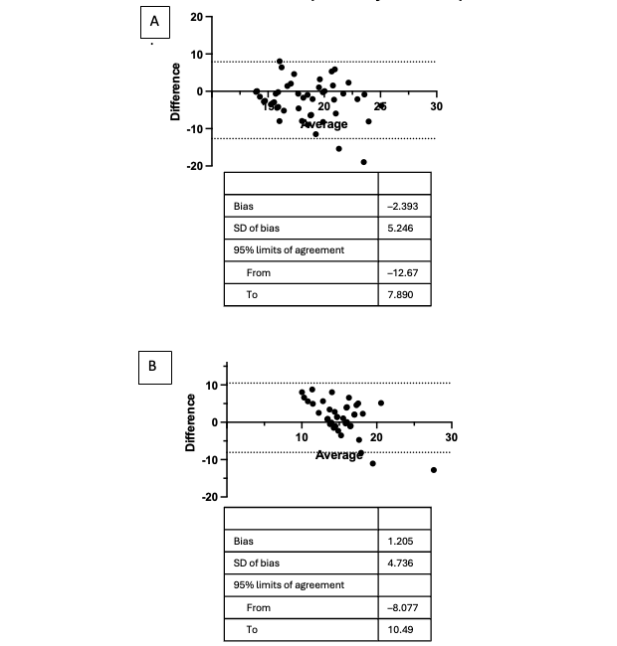
Bland-Altman plot of (A) cardiopulmonary and (B) noncardiopulmonary chief complaint.

Participants grouped by cardiopulmonary chief concern plotted on a Bland-Altman analysis showed a bias of –2.39 (Figure 4). The upper limit of agreement was 7.89, and the lower limit of agreement was –12.67. Participants without a cardiopulmonary chief concern showed a Bland-Altman bias of 1.21 with an upper limit of agreement of 10.49 and a lower limit of agreement of –8.08. While there was no clear trend for the cardiopulmonary chief concerns, the noncardiopulmonary chief concern measurements followed a similar trend to the overall Bland-Altman, with the bias being more positive at lower respiratory rates and more negative at higher respiratory rates. There was not a statistically significant correlation for either the cardiopulmonary or noncardiopulmonary chief concerns. The ICC was 0.21 (–0.41 to 0.56) and 0.42 (–0.09 to 0.69) respectively.

**Figure 5:**
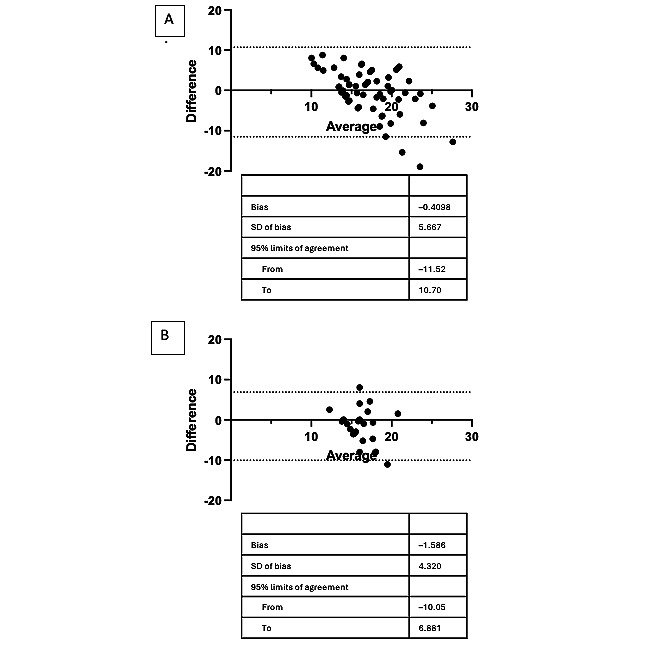
Bland-Altman plot for (A) male and (B) female candidates.

When grouped by sex, the Bland-Altman analysis had a bias of –0.41 (–11.56 to 10.70) for male candidates and a bias of –1.52 (–10.05 to 6.881) for female candidates (Figure 5). The overall trend for male candidates was also positive at lower respiratory rates and negative at higher respiratory rate; however, there was no clear trend for the female candidates. There was no statistically significant correlation for either the male or female candidates. The ICC for male candidates was 0.45 (0.08-0.67) and for female candidates was –0.40 (-2.13-0.47).

## Discussion

### Overview

Although overall there was no statistically significant correlation, the P440 agreed best within the physiologically normal respiratory range. There was less agreement at both increased and decreased respiratory rates as the P440 was underestimated at higher respiratory rates and overestimated at lower respiratory rates. One possible reason for not achieving statistical agreement is that fewer participants had episodes of bradypnea (slow respiratory rate) or tachypnea (high respiratory rate) during the study period and therefore the measurements were more prone to error. Additionally, the ED is a busy environment, and there were many events that could have affected the accurate measurement of respiratory rate, including patient movement, patient clothing or obstruction by blankets, people entering and exiting the room, and the need for bedside procedures. Understanding this trend is important to adjust future respiratory models to be more accurate in the abnormal ranges. However, further validation will be needed before it is able to be used in clinical settings.

There have been other recent studies using an ultrawideband to detect respiratory rates [17,18]. He et al [17] used a combination of a 3D depth camera and ultrawideband radar to use localization with the radar technology to detect respiratory rate in an experimental lab setting. However, the results were limited when movement (such as a person walking) interfered with the respiratory signal. Lauteslager et al [18] also used a ultrawideband radar to detect respiratory rates in a variety of controlled clinical settings. The radar was able to accurately detect respiratory rate; however, the study excluded time periods of irregular respiratory patterns, apnea, and high-motion artifacts. While lab and controlled patient scenarios are important for the initial testing of a monitor, it is also necessary to understand how the device performs in real-world settings where there is unplanned motion and noise. Additionally, studying the device in a variety of clinical scenarios, such as patients with invasive mechanical ventilation, may help overcome some of the real-world barriers and understand the setting in which the device best performs. By piloting the P440 in the ED, we were able to gather a better understanding of how the device will perform in an uncontrolled setting.

There are several limitations to this study. First, this was a small study. This was designed to be a pilot study, and therefore a convenience sample was obtained during the enrollment period. Future studies will aim to recruit additional participants in order to validate the P440. Additionally, there are difficulties inherent in measuring respiratory rates that may have affected the accuracy of the P440. Gold standard respiratory rate monitoring requires direct observation that may not only affect a participant’s breathing pattern but also necessitate close proximity, which can cause interference with the monitor. Motion artifacts are also known to cause inaccuracies in respiratory monitoring, which is similarly seen in the standard of care telemetry monitoring that is currently used in practice.

Future iterations of the contactless monitoring system will focus on improving the respiratory rate algorithm. To achieve this, we plan to investigate the Eulerian phase magnification of the radargram signal in order to extract meaningful features [19]. The Eulerian phase magnification approach was first introduced for video magnification; however, its use for human motion estimation was limited by privacy concerns. Using the ultrawideband radar with this approach may provide a convenient, nonrestrictive, and unobtrusive means to detect motion, especially in noisy conditions like the ED. Our next step is to build a 1D radar signal magnification pipeline using the Eulerian phase-based magnification algorithm’s complex Gabor wavelet pyramid [20,21]. Different spatial wavelengths of the Gabor pyramid will give us different 1D signals from which we will get FFT peaks and zero-crossing rates as features. Using these informative features, we plan to fit a regression model to better estimate the respiratory rate. The motivation behind using a motion magnification-based algorithm is to leverage the amplification of subtle motions in a dynamic environment that might be difficult to observe by conventional temporal models. Additionally, by using machine learning models, we can get a better respiratory rate estimate by generalizing across different settings as well as diverse demographics.

Overall, the contactless radar respiratory monitor provides a unique solution to monitor patients in places that previously presented challenges, such as low-acuity outpatient settings, waiting rooms, and the home. The ability to monitor changes in respiratory rate accurately and continuously in these settings can help detect clinical deterioration without having direct contact with the person. This has become increasingly important, especially during the COVID-19 pandemic and with the increase in hospital crowding and long ED wait times.

### Conclusions

This pilot study has provided important preliminary data that will be used to inform the development of future iterations of the P440 respiratory rate model. Once validated, the device can be miniaturized and used in a variety of settings to provide continuous respiratory monitoring that can be remotely accessed to detect clinical changes in a variety of disease states and improve the care of patients.

## Abbreviations

ED: emergency department
FFT: fast Fourier transform
ICA: independent component analysis
ICC: intraclass correlation coefficient
P440: PulsON 440
UNIX: Uniplexed Information Computing System

## Data Availability

The data sets generated during and analyzed during this study are available from the corresponding author on reasonable request.
